# Femtosecond Laser Microfabrication of an Integrated Device for Optical Release and Sensing of Bioactive Compounds

**DOI:** 10.3390/s8106595

**Published:** 2008-10-23

**Authors:** Diego Ghezzi, Rebeca Martinez Vazquez, Roberto Osellame, Flavia Valtorta, Alessandra Pedrocchi, Giuseppe Della Valle, Roberta Ramponi, Giancarlo Ferrigno, Giulio Cerullo

**Affiliations:** 1 NeuroEngineering and Medical Robotics Laboratory, Bioengineering Department, Politecnico di Milano, 20133 Milano, Italy. E-Mails: diego.ghezzi@iit.it; alessandra.pedrocchi@polimi.it; giancarlo.ferrigno@polimi.it; 2 Department of Neuroscience and Brain Technologies, The Italian Institute of Technology, 16163 Genova, Italy; 3 Istituto di Fotonica e Nanotecnologie (IFN) - CNR, Dipartimento di Fisica, Politecnico di Milano, 20133 Milano, Italy. E-Mails: rebeca.martinez@polimi.it; giuseppe.dellavalle@polimi.it; roberta.ramponi@polimi.it; giulio.cerullo@fisi.polimi.it; 4 San Raffaele Scientific Institute and Vita-Salute University, 20132 Milano, Italy. E-Mail: flavia.valtorta@hsr.it; 5 Unit of Molecular Neuroscience, The Italian Institute of Technology, 20132 Milano, Italy

**Keywords:** Caged compound, waveguides, laser uncaging, optical release, femtosecond microfabrication, optical sensing

## Abstract

Flash photolysis of caged compounds is one of the most powerful approaches to investigate the dynamic response of living cells. Monolithically integrated devices suitable for optical uncaging are in great demand since they greatly simplify the experiments and allow their automation. Here we demonstrate the fabrication of an integrated bio-photonic device for the optical release of caged compounds. Such a device is fabricated using femtosecond laser micromachining of a glass substrate. More in detail, femtosecond lasers are used both to cut the substrate in order to create a pit for cell growth and to inscribe optical waveguides for spatially selective uncaging of the compounds present in the culture medium. The operation of this monolithic bio-photonic device is tested using both free and caged fluorescent compounds to probe its capability of multipoint release and optical sensing. Application of this device to the study of neuronal network activity can be envisaged.

## 1. Introduction

The local application of bioactive molecules is one of the most suitable approaches to study the dynamic responses of living cells in order to understand the molecular mechanisms underlying their physiological functioning. To this purpose, flash photolysis of caged compounds has been intensively used to quickly release bioactive molecules in spatially controlled regions [1]. Caged compounds are biological molecules (e.g. neurotransmitters, ions, fluorochromes, peptides and enzymes) inactivated by the presence of a blocking chemical group, typically a nitrobenzyl group. Such compounds can be optically activated by photo-cleaving the protecting group using high energy ultra-violet (UV) radiation in a window generally ranging from 340 to 380 nm [2].

Thus far, various approaches have been adopted in order to obtain optical release. Traditionally, local activation of caged compounds has been performed by using a high power UV incoherent light source, such as a xenon or a mercury fluorescence arc lamp, and an electro-mechanical shutter to produce short flashes delivered to the sample through the objective lens of an epi-fluorescence microscope. Alternatively, flash lamps have been used [3], but if combined with electrophysiological recording techniques, they produce large electromagnetic artifacts and require a considerable amount of time to be re-charged. Another possible solution is based on the combination of a laser and a confocal [4] or two-photon [5] microscope, to allow precise local uncaging of both *in vitro* and *in vivo* experiments. These approaches require a high numerical aperture objective in order to achieve a strongly localized stimulation, sacrificing the wide-field analysis of the sample after the stimulation.

Recently, alternative strategies based on more flexible and cost effective solutions have been also proposed to uncage compounds. The use of optical fibers coupled to arc lamps allows to spatially localize the photoactivation of the compounds by reducing the fiber's tips [6] and to simplify the positioning task by moving the optical fiber with a micro-manipulator [7]. Low cost excitation sources for flash photolysis have been used, such as nitrogen lasers [8] and UV light-emitting diodes [9].

In the last two years, new efforts have been devoted to the design of integrated solutions for the optical stimulation tool. Arrays of semiconductor ultra-violet light-emitting diodes [10] have been placed under a neuronal culture for the optical stimulation. In the same year a device based on an optical fiber bundle has been proposed [11] to stimulate neurons cultured over a Micro-Electrode Array device. In both cases the stimulation tool has not been integrated in the substrate containing the living cells. Thus, a monolithically integrated solution is still highly requested.

Femtosecond lasers have rapidly become powerful, flexible and reliable tools for micromachining of transparent materials. The nonlinear absorption, induced by the very high peak intensity achieved in the pulse focus, allows delivering energy inside the sample in a highly controlled and spatially localized way. This unique feature of femtosecond laser micromachining enables three-dimensional structuring of the material with a direct and maskless process. According to the pulse energy, laser repetition rate and focusing optics several micromachining functions can be performed with femtosecond lasers. A vast literature is already available on optical waveguide writing in glasses and crystals [12-16]. Recently, drilling [17] and welding [18] of glasses have also been proven. In addition, irradiated regions show a preferential etching rate when the fused silica substrate is immersed in aqueous solutions of hydrofluoric acid (HF), allowing the fabrication of microchannels [19] and deep cuts in such glass. These different capabilities have seldom been combined, but could make femtosecond lasers an all-in-one tool for the complete fabrication of optical devices and in particular of bio-photonic devices [20, 21].

**Figure 1.**
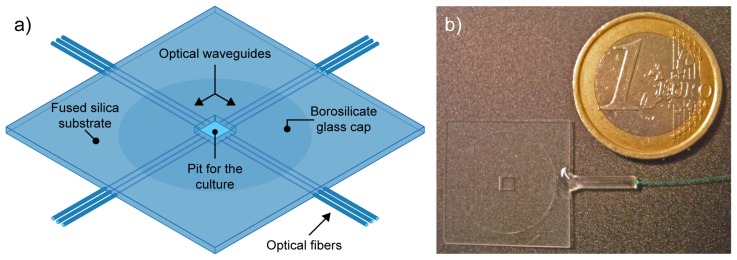
(a) Rationale of the chip for optical uncaging. Several optical waveguides drive excitation light in to the cell culture region (central well). The culture will take place on a thin glass, allowing high quality optical imaging from the bottom. (b) Top view of the fabricated chip. One of the waveguides was bonded to an optical fiber.

Here we demonstrate the use of femtosecond laser radiation for the microfabrication of an integrated device to optically probe the dynamic response of living cells. The cutting capabilities are used to create a pit in a glass sample where the cells can grow; optical waveguides are then directly inscribed in the glass sample for a multipoint and monolithical addressing of the culture in the pit. The advantage of this approach is the complete absence of any micromanipulation of optical elements by the end-user with the significant possibility of automating the measurements. The rationale of the chip for optical uncaging is illustrated in Figure 1(a). It is based on a 500-μm-thick fused silica substrate with a 2 mm-square well in the center, representing the region for the cell culture.

This well is closed by a 180-μm-thick borosilicate coverslip, previously glued on the bottom surface of the chip. In this way, the culture is grown on a thin glass allowing to achieve high quality optical imaging with oil-immersion objectives. The square well is then optically addressed from the sides by several optical waveguides, each bonded to an optical fiber which is in turn connected to the excitation laser source for optical uncaging in different positions of the well. In the following sections we will describe how such device has been fabricated by using only femtosecond lasers as microfabrication tools. In addition, preliminary characterization of its operation will be given for monolithic optical uncaging.

## 2. Chip fabrication

**Figure 2.**
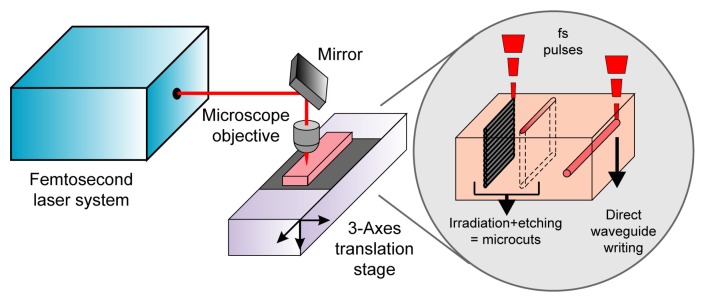
Schematic set-up for femtosecond laser micromachining. Multilevel irradiation followed by selective chemical etching is used to perform microcuts through the whole sample thickness. Direct waveguide writing can also be performed by using a lower intensity irradiation.

The experimental setup used for the fabrication of the device is shown schematically in Figure 2. It is based on femtosecond laser radiation focused by a microscope objective inside the fused silica substrate (Lithosil, Schott AG, Germany), which is suitably moved by 3D computer-controlled translation stages (Physik Instrumente, Germany). The femtosecond laser micromachining capabilities used in the present work are the fabrication of microcuts through the glass slab and the direct writing of optical waveguides (see inset of Figure 2). Two different lasers were used, one for fabricating the microcuts and a second one for writing the optical waveguides. For the microcutting we used a diodepumped cavity-dumped Yb:KYW laser, generating 350-fs pulses at 1,030-nm wavelength with pulse energies up to 1 μJ at a repetition rate of 600 kHz. The waveguide fabrication was performed by means of a regeneratively amplified Ti:sapphire laser generating 150-fs, 500-μJ pulses at 1 kHz and 800 nm. Two different lasers were used because at present the best waveguides in fused silica are fabricated with the low repetition rate Ti:sapphire laser [20], while for the multilevel irradiation, necessary to obtain microcuts, the high repetition rate Yb:KYW laser provides much higher processing speed; the latter laser, however, is much more compact, reliable and suited for industrial environment, therefore work is currently in progress on improving the quality of the waveguides written with this laser. Indeed, the final aim is the use of a single laser for all the micromachining operations.

**Figure 3.**
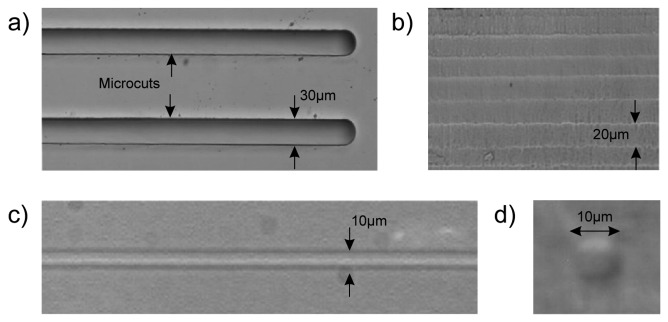
Microscope pictures of: (a) fused silica substrate with two cuts made by irradiation and subsequent etching in HF, (b) vertical wall of one of these cuts, (c) top view of an optical waveguide written with the femtosecond laser inside the substrate and (d) end view of the waveguide.

The microcuts are fabricated by a two-step process: femtosecond laser irradiation followed by chemical etching in HF solution. For the irradiation the laser beam is focused inside the sample by a 0.3 numerical aperture (NA) 20× microscope objective and the sample is translated along directions transversal to the beam path, with a speed of 1mm/s. The scanning of the laser focus produces exposed areas while the surrounding material remains unaltered. The scanning is repeated multiple times at different depths (see Figure 2), creating volumes of exposed material that present etching rates higher than the unexposed regions; this allows their selective removal by immersing the sample in a 20% HF solution. In this way, deep cuts in the fused silica substrates are fabricated. Figure 3(a) shows the microscope images of some parallel cuts, created with this technique, and Figure 3(b) the vertical surface of one of their inner walls. It is worth noting that the surface optical quality is good without any further processing even if several lines are visible due to the multilevel irradiation.

The demonstrator chip is fabricated in a 2 cm-square fused silica substrate with a thickness of 500 μm. To produce the central pit, squares of 2 mm side are written inside the substrate separated by 20 μm in depth until covering the whole thickness. Then the substrate is immersed in a 10% HF aqueous solution for 3 hours, in order to separate the square central part. To create a floor in the central well, where a cell culture will take place, the bottom of the hole is closed by gluing a circular borosilicate coverslip with a 13 mm diameter and an 180 μm thickness. In this way, high quality images of cultured cells can be acquired through the thin glass with oil-immersion objectives. The coverslip is glued to the fused silica by a clear liquid photopolymer (Optical adhesive 61, Norland Products Inc., USA) that is cured with UV light. When fully cured this adhesive provides very good adhesion and high solvent resistance, which allows one to manipulate the chip and clean it before making the cell culture.

Optical waveguides are written focusing the laser beam by the same objective (NA 0.3) and translating the sample, transversally, at a speed of 20 μm/s. In order to produce waveguides with a circular cross section, the beam is astigmatically shaped by passing it through a cylindrical telescope [15]. All waveguides have been written near to the bottom of the sample (from 10 to 20 μm) to ensure that guided light reaches cells placed on the floor of the pit. A microscope image of one of these waveguides with a diameter of about 10 μm is shown in Figure 3(c) (top view) and Figure 3(d) (end view). An array of ten waveguides was fabricated on each side of the chip to demonstrate that, with this device, it is possible to achieve a multipoint excitation of the cell culture inside the pit. One of these waveguides was bonded to an optical fiber to allow easy and reproducible light-coupling and to demonstrate the possible compact and alignment-free layout of the final device. A picture of the demonstrator is shown in Figure 1(b), where its small dimensions are compared to those of a coin.

## 3. Chip validation

**Figure 4.**
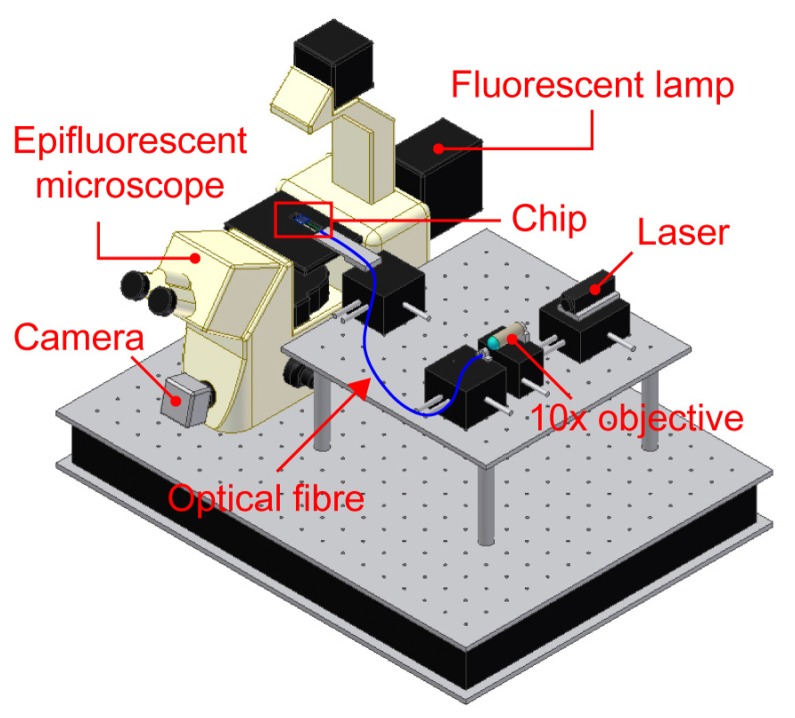
Schematic view of the experimental set-up for fluorescence experiments. The laser light is coupled to the optical waveguides in the chip through an optical fiber. Fluorescence images of the chip are taken with a dedicated microscope.

In order to validate the chip operation two different tests were performed, involving the activation of both free and caged fluorescent compounds. The setup employed to perform these experiments is schematically shown in Figure 4. A laser radiation is coupled to an optical fiber using a 10× objective and then launched into the selected optical waveguide. In our experiments the optical fiber was either bonded to the chip or juxtaposed to it using a dedicated micro-manipulator (Melles Griot, USA). This last option was used in order to serially excite different waveguides; however, the final configuration will implement a dedicated fiber bonded to each waveguide. Images of the fluorescence were taken using a microscope equipped with a high sensitivity camera.

**Figure 5.**
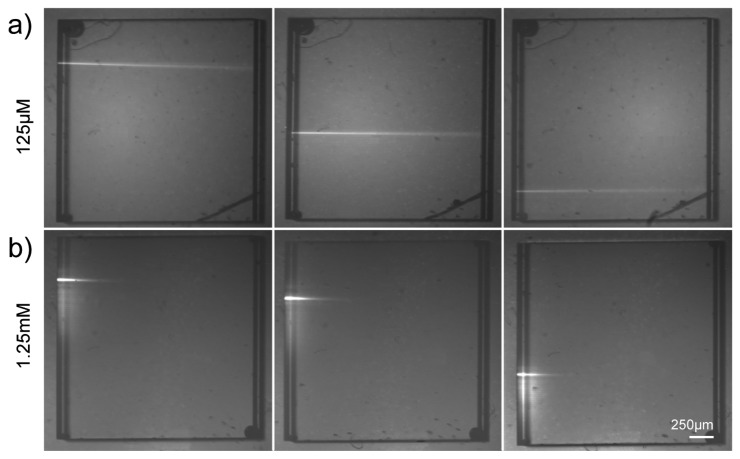
Excitation of a solution of Rhodamine 6G in methanol at two different concentrations: (a) 125 μM (b) 1.25 mM. Three optical waveguides on the left side of the chip are alternatively used. The scale bar is 250μm for all panels.

The first experiment aimed at demonstrating the capability of the optical waveguides to deliver light with a high spatial selectivity to the content of the square well at the center of the device. To this purpose the well was filled with a solution of Rhodamine 6G (Exciton, USA) in methanol at two different concentrations, 125 μM and 1.25 mM, respectively. The optical waveguides were used to locally deliver the light needed for the fluorescence excitation. In order to image the induced fluorescence, the chip was mounted under a stereomicroscope (MZ12.5, Leica Microsystems, Germany) equipped with a CCD camera (XC-ST70CE, Sony, Japan). A green He-Ne laser source at λ = 543 nm (Melles Griot, USA) was coupled to the optical waveguides on the left side of the chip through the optical fiber. The images of the fluorescence excited through the waveguide are shown in Figure 5 with a suitable long-pass color filter (OG570, Schott Inc., Germany) in detection. The top panel [Figure 5(a)] shows the excitation of the 125 μM Rhodamine 6G solution by three optical waveguides alternatively coupled. It can be appreciated that the waveguides provide a very selective excitation thus showing the possibility of multipoint stimulation allowed by the chip. At this concentration level the fluorescence was excited by the light coming from the waveguide along the whole well. Due to the long Rayleigh range (z_R_ = 520 μm) of the waveguide mode (waist w_0_ = 8 μm) the excitation remains spatially confined with limited divergence.

Figure 5(b) shows the results with a 1.25 mM Rhodamine 6G solution. Again the possibility of multipoint stimulation was demonstrated, but in this case the fluorescence was generated only in a small strip near the waveguide output. The shortening of the excited area is due to the increase of the probe concentration indicating an inverse relationship between the absorption length and the concentration of the chromophore in the sample, according to Lambert-Beer's law.

The second experiment aimed at demonstrating that the waveguides could also be used to locally release caged molecules, exploiting the laser light from the waveguide to activate compounds with high spatial resolution. A caged compound fluorescein bis-(5-carboxymethoxy-2-nitrobenzyl) ether, dipotassium salt (CMNB-caged fluorescein) from Invitrogen (Italy) was used. Photoactivation of this compound generates a fluorescein derivate with spectral characteristic similar to 5-carboxyfluorescein (5-FAM).

To perform the experiment the central well of the chip was filled with a 100-μM CMNB-caged fluorescein solution in glycerol. A UV laser source at λ = 375 nm (BCL-09-375, Crystal Laser, USA) was used to deliver millisecond pulses to the sample via the bonded waveguide at the right side of the chip. The chip was mounted on an inverted epifluorescent microscope (Axiovert 200, Zeiss, Germany) equipped with a 5× Plan-Neofluar objective, a metal-halide fluorescence illuminator (x-cite 120, EXFO, Canada) and a CMOS intensified camera (Focuscope SV-200i, Photron Limited, Japan), this allowed us to take fluorescence images through a dedicated filter set (BP 450/490, FT 510, BP 515/565). To evaluate the photoactivation induced by the laser light guided through the waveguide, the whole well was illuminated by the excitation lamp and fluorescence images were taken before, during and after the laser pulse.

**Figure 6.**
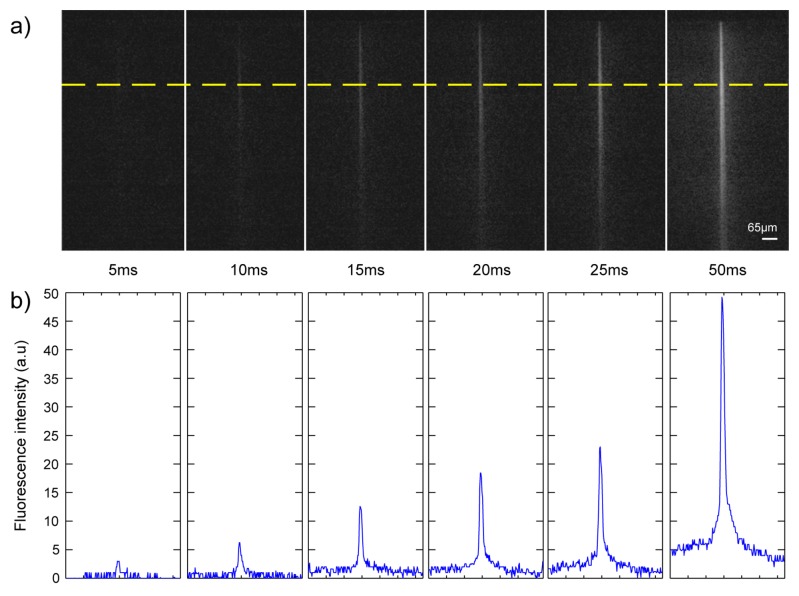
Optical uncaging of a caged fluorescent compound. **(a)** Fluorescence pictures of the activated region for six different pulse durations. **(b)** Transversal profile of the fluorescence intensity along dashed line for different excitation energies. Scale bar is 65μm for all panels.

The fluorescence images of the well (see Figure 6) prove that the optical uncaging of CMNB-caged fluorescein was successfully achieved with the UV light coming out of the waveguide, demonstrating the capability of this chip to activate compounds in localized regions inside the well. The top panel [Figure 6(a)] shows the fluorescence arising from the uncaged region for six different pulse durations of the UV laser: 5, 10, 15, 20, 25 and 50 ms. The pictures represent a single frame acquired 15 ms after application of the stimulus. As evident, the fluorescence intensity increases with the pulsewidth of the stimulus becoming clearly visible for pulses longer than 15 ms. The growth in the fluorescence intensity emitted from the sample with the pulse duration indicates that the number of uncaged molecules increases with the energy delivered. This effect is also quantified in Figure 6(b) showing a line profile measured along the yellow line in Figure 6(a). Finally, it can be appreciated that the width of the activated area remains comparable to the dimension of the waveguide section in the transversal direction. Therefore, by inscribing arrays of waveguides on the four sides of the well it should become possible to create a light-grid to uncage compounds in spatially controlled regions of the well.

## 4. Conclusions

A monolithic device for multipoint and spatially selective optical uncaging of bioactive compounds has been demonstrated. The device has been completely fabricated using femtosecond lasers as micromachining tools. Such lasers allowed both the fabrication of the pit, where cell culture is to be performed, and the inscription of optical waveguides for photoactivation of caged compounds. The chip has been experimentally validated demonstrating its capability to address different regions of the sample and the possibility to control the length of the excited region by the duration of the excitation pulse.

This new approach gives all the advantages allowed by the use of monolithic bio-chips, including the automation and the absence of manipulated optical elements. However, some issues can be pointed out with respect to the other non-monolithic available techniques. In fact traditional external devices (e.g. optical fibers, microscope objectives, galvanometers) employed for upright or inverted optical uncaging provide more localized stimulation point, typically a spot instead of a line. This feature intrinsically produces a great improvement in the spatial resolution during an uncaging task performed with cultured dissociated cell. With the aim to close this discrepancy, our efforts are now dedicated to upgrade our stimulation profile developing a new chip with skew waveguides, allowing spot stimulation of the culture laying at the bottom of the pit.

The results obtained in this work pave the way to the application of this bio-chip to the study of the dynamic cell responses. Experiments focused on the evaluation of the neuronal network responses induced by photo-uncaging of neurotransmitters glutamate and monitored through calcium indicators are currently under investigation.
